# Nurses’ interactions shape coparenting relationships during early parenthood: a longitudinal study of fathers with infants in Sweden

**DOI:** 10.1186/s12912-025-03508-9

**Published:** 2025-10-01

**Authors:** F. Kübra Aytaç-DiCarlo, Sarah J. Schoppe-Sullivan, Michael B. Wells

**Affiliations:** 1 Department of Psychology, The Ohio State University, Columbus, USA; 2 Women’s and Children’s Health, Karolinska Institutet, Stockholm, Sweden

**Keywords:** Child health nurses, Coparenting, Father involvement, Early parenthood, Child health centers, Longitudinal study, Sweden, Parenting support, Family well-being, Responsive communication

## Abstract

**Background:**

During the transition to parenthood, the involvement of fathers in childrearing and coparenting is crucial for the well-being of the family. Nurses play a significant role in supporting fathers, but little is known about the practical support they provide in asking and answering questions. This study longitudinally investigates the impact of nurses’ interactions with fathers of infants on the coparenting relationship during infancy and toddlerhood.

**Methods:**

Data were collected from the Pappor/Icke-Födande Föräldrar study, involving 413 fathers in Region Stockholm, Sweden, who attended the 3-5-month child health center (CHC) visit. Surveys were administered at baseline (infant mean age in months *M* = 9.11 and Min–Max = 1–23), 6-month, and 18-month follow-ups. The study measured the extent to which nurses answered and asked fathers’ questions and the quality of coparenting relationship was measured using the Brief Coparenting Relationship Scale. Multiple imputation was used for missing data and path analysis was conducted to assess the effects.

**Results:**

Nurses fulfilled 79% of items related to answering fathers’ questions and 58% of items related to asking questions. Answering fathers’ questions significantly improved coparenting relationships at baseline (*β* = 0.22, *p* < .001) and at the 6-month follow-up (*β* = 0.06, *p* < .01). However, the effect of asking questions was less pronounced. Coparenting relationships declined significantly at the 18-month follow-up.

**Conclusions:**

Nurses’ responsive communication during early parental visits positively impacts fathers’ coparenting relationships in the short to intermediate term. However, sustained coparenting quality over time may require continuous support and follow-ups. Enhancing training and resources for nurses to balance reactive and proactive interactions is crucial for fostering positive coparenting interactions and family health.

**Clinical trial number:**

Not applicable.

**Supplementary Information:**

The online version contains supplementary material available at 10.1186/s12912-025-03508-9.

## Introduction

During the transition to parenthood (TTP), the couple’s relationship undergoes a significant transformation, evolving into a more complex family structure[1]. This shift marks a departure from the dyadic couple relationship, to include the roles and interactions related to their new parental responsibilities. The couple’s dynamic now encompasses a new layer of interaction and responsibility centered on their child, which gives rise to what is known as a coparenting system [2]. The essence of coparenting lies in how parents interact with each other regarding their children. This includes how they coordinate their parenting strategies, communicate about their child’s needs, and provide support and guidance to each other in their parenting roles [3].

Considering that early patterns of parental interactions are indicators of how coparenting dynamics evolve into the preschool years and difficulties in coparenting during the first year of parenthood can predict later adjustment problems in children [4–6], understanding how to best support and strengthen these initial coparenting dynamics is crucial. Despite increasing attention to fathers’ involvement and coparenting during the perinatal period, there remains a lack of research on specific, concrete clinical practices that support fathers’ participation which can enhance the coparenting relationship. Addressing this gap is especially important in the context of Sweden’s progressive parental leave and child health care systems, where policy frameworks promote equal support for both parents, but further work is needed to translate these intentions consistently into practice.

### Impact of coparenting on child and family well-being

Effective coparenting involves mutual support and shared responsibilities, enabling parents to address their child’s developmental needs collaboratively. When parents engage in coparenting with a high degree of cooperation and low levels of conflict, it positively influences the child’s socio-emotional, cognitive, and developmental outcomes [7, 8]. In contrast, coparenting conflicts are linked to higher risks of externalizing and internalizing issues and insecure attachment in children [9]. Even when considering the impact of family dynamics and each parent’s relationship with their child, the associations between coparenting and child outcomes remain strong (e.g., [10, 11]). Thus, the transition to parenthood not only redefines the couple’s relationship but also establishes a new framework for interaction and cooperation within the family system. This framework is crucial for creating a supportive environment that benefits both the parents and their children [12, 13].

Successful coparents effectively collaborate, communicate, share expectations, and maintain a clear and consistent family structure with defined boundaries between parents and children. They guide their children in alignment with family goals, while supporting each other’s well-being. Research indicates that while coparenting is deeply influenced by early parenting experiences, some suggest it begins before the child’s birth [14, 15], whereas others argue that it practically starts after the child is born [2, 16]. Research emphasizes the significance of the TTP for paternal involvement, as fathers’ involvement during pregnancy and birth is linked to their engagement with their children after birth [17, 18].

### Role of perinatal clinical care in supporting father involvement and coparenting

Perinatal clinical care that focuses on the coparenting relationship has the potential to improve paternal outcomes [19]. Clinicians including midwives and child health nurses, referred to hereafter as nurses, frequently interact with expectant and new parents as part of their routine duties, making these interactions potentially beneficial for fathers [20–23]. Fathers are more motivated to attend visits when they are specifically invited by the nurse [24], and they want to gain knowledge from the nurse to better support their child, have the opportunity to ask questions and express concerns, and to physically interact with the nurse [25, 26], leading to increased parental efficacy and decreased stress [27]. Since parenting self-efficacy can impact the coparenting relationship [28], nurses’ support of fathers might be particularly beneficial. Wells, Gedaly, and Aronson (2021) found that attendance at CHCs and total support from nurses was associated with more positive coparenting relationships. Therefore, Xiao and Loke’s [29] literature review recommends fathers to receive prenatal and postnatal clinical support to enhance the coparenting relationship. However, much more research is still needed in this field to better understand the impact clinicians have on fathers’ coparenting relationships [19].

Nurses generally do not see it as their primary role to encourage father involvement, and only around half of nurses believe that fathers are as sensitive as mothers to infants’ needs [30]. Although there are subtle differences in parenting styles between mothers and fathers, evidence indicates that sensitivity and responsiveness are more strongly associated with caregiving experiences and psychological factors rather than parental sex [31]. Fathers tend to interact with newborns similarly to mothers, including experiencing synchronized hormonal changes [32]. Thus, fathers need to be given the opportunity and support by nurses to better navigate the TTP.

### Swedish family policies and child health centers

In Sweden, as in many other countries, mothers have traditionally been responsible for childcare, despite gender roles being highly equal compared to other countries [33]. However, over recent decades, fathers have progressively become more involved in childcare (see [34]), as social policies supporting gender equality, such as providing equal access to parental support for both mothers and fathers, generous parental leave, and child allowance in addition to an extra two weeks of paternity leave for fathers, have been societally promoted [35–37].

Moreover, the child health centers (CHCs) have evolved over the past 80 years in Sweden. CHCs aim to assist parents during their TTP, provide immunizations, monitor children’s physical, social, and emotional development, and offer guidance on breastfeeding, weaning, and general childrearing [38]. In Sweden, the CHCs are provided free of charge and are available to all parents from the birth of the child until the child is six years old. Participation is voluntary, with nearly 100% of Swedish children enrolled, reflecting high trust in the system [38]. To promote greater involvement of fathers and provide tailored support, Region Stockholm began offering an individual conversation for the father or non-birthing parent when the child was 3 to 5 months old. Introduced in 2017, this visit was initially implemented on a voluntary basis, with approximately 65% of nurses in the region choosing to participate in the training and offer the visit [39]. These guidelines were then implemented nationally in 2019 [40]. During this visit, a child health nurse discusses several topics with the father, including the child’s growth and development, the father’s relationship with the child, his own mental health, and the dynamics of the coparenting relationship.The national guidelines aim for equal involvement of both parents in CHC services, but achieving this shift is challenging and depends on changing practitioners’ beliefs and overcoming perceived barriers.

Nurses believe that the visit promotes fathers and other non-birthing parents’ participation, which can help strengthen the CHCs’ family-oriented care, and thus further help to improve family health [41]. Facilitators of support for fathers included nurses actively engaging fathers, including personally inviting them to the 3–5 month visit, adapting to their needs, and fostering a supportive environment, while barriers included financial constraints and the high workload of nurses, which made it challenging to implement the visits effectively [42]. However, nearly one-third of nurses did not adhere to following the national guidelines [39]. In addition, nurses also reported barriers to implementing the visits including financial constraints and having a high workload [42], as well as cultural and language barriers, and societal norms [41], which affected fathers’ participation. Additionally, nurses report difficulties in reaching fathers and socially vulnerable groups, but have not implemented specific strategies to address these challenges [43].

In this context, nurses’ roles extend beyond just providing care to also potentially acting as gatekeepers in the family’s caregiving experience. While nurses are instrumental in supporting parents and ensuring effective infant care, their approach can influence how involved fathers feel and how well they connect with the care process. As such, the way nurses engage with fathers—such as through supportive, gateopening behaviors—can significantly impact paternal involvement and the overall quality of family-centered care. One specific way to display gateopening behaviors and be supportive is to ask and answer fathers’ questions during the visits. The present study therefore investigates if, when, and to what extent fathers’ coparenting relationships are affected by nurses’ answering fathers’ questions and by nurses’ asking fathers questions from the father/non-birthing parent visit guidelines when the infant is three-to-five months old. By focusing on these actionable clinical interactions, this study aims to shed light on how routine healthcare practices can be leveraged to promote coparenting quality during the early postnatal months.

## Method

### Data

Data were retrieved from the Pappor/Icke-Födande Föräldrar study (PIFF; Fathers/Non-birthing Parents Study), which distributed surveys to fathers of infants and toddlers in Region Stockholm, Sweden. Fathers in the study participated in up to three waves of data collection between 2018 and 2022: at baseline, at a 6-month follow-up, and at an 18-month follow-up. To provide results that are representative of fathers’ experiences, the current study sample only included those fathers who attended the 3-5-month visit (*n* = 413) and not the total sample of fathers included in the PIFF study (*n* = 922).

Table 1 presents a comparison of the study sample and the total sample across all variables. The comparison indicates that the samples only differed significantly in relation to the nurses’ invitation of the father to the 3-5-month visit (*p* < .001) and the infant’s/toddler’s age at baseline (*p* < .001). Therefore, fathers’ perceptions of nurses inviting them to attend leads to greater attendance at the 3–5 month visit. Infants were younger in the total sample, since to attend the 3–5 month visit, infants needed to be at least three months old.

**Table 1:** Descriptive statistics comparing the study sample to the total sample

	Study sample (*n* = 413)							Total sample (*n* = 922)			
	M	SD	Quartiles	Freq.	%	M	SD	Quartiles	Freq.	%	Effect size
Father’s age (years)	34.54	5.72				34.43	5.44				0.20
Infant’s age (months)											0.08
First quartile			6					4			
Second quartile (median)			8.5					7			
Third quartile			11					11			
Household income per month (SEK)											0.03
First quartile			45,000–54,999					45,000–54,999			
Second quartile (median)			65,000–74,999					75,000–84,999			
Third quartile			85,000–94,999					85,000–94,999			
Father’s number of children	1.46	0.76				1.52	0.76				0.08
Father’s origin											0.45
Native				359	87.1				812	88.2	
Foreign				53	12.9				109	11.8	
Pregnancy planning											0.20
Yes				341	83.4				755	82.9	
No				68	16.6				156	17.1	
Nurse invited the father to the 3-5-month visit											0.16***
Yes				274	66.8				389	45.3	
No				136	33.2				469	54.7	
Nurse has answered questions	0.79	0.21				0.79	0.21				^a^
Nurse has asked questions	0.58	0.27				0.58	0.27				^a^
Coparenting relationship Time 1	59.89	10.40				59.86	10.68				0.09
Coparenting relationship Time 2	59.29	9.64				59.62	9.09				0.02
Coparenting relationship Time 3	53.81	6.19				53.52	6.27				0.01

Note. The descriptive statistics relate to the non-imputed datasets at baseline (Time 1). The study sample includes the fathers who attended the 3-5-month visit (for all of whom this visit had been implemented), and the total sample includes all fathers who participated in the data collection at Time 1. The effect sizes indicate differences between the samples across all variables. The effect sizes include phi (for the binary variables), Cramer’s V (for the ordinal variables presented with quartiles), and Cohen’s d (for the continuous variables presented with means)

^a^ The mean values for the nurses’ answering questions and asking questions were identical in the study sample and the total sample because they could only be calculated for participants with complete data, all of whom were included in both samples

*** *p* < .001

### Procedure and ethics

Fathers were recruited through paid Facebook advertisements that featured the university’s name and a question and a statement about their parenting: Advertisement #1: “Are you a new father and satisfied with your new life as a parent?” or Advertisement #2: “Are you struggling with your parental role?”. Both advertisements then concluded with: “Participate in our survey on the father visits at the child health centers (CHCs)”. Below this sentence was an image showing either a satisfied or struggling father-infant interaction. Participants were recruited through advertisements, which ran from November to December 2018 and from November 2019 to March 2020. Upon clicking the advertisements, fathers were directed to the web-based survey, where this landing page provided potential participants with an information letter about the study, including a link to more detailed information located on a university website, and advised that by clicking “next” they were consenting. The current study was granted ethical approval by the Stockholm Regional Ethics Board (dnr: 2018/889 − 31/5). Eligibility criteria included identifying as: (i) fathers of an (ii) infant (aged 0 to 24 months) and (iii) having sufficient Swedish-language skills to complete the survey. Fathers did not receive any direct incentives for completing the survey.

### Measures

#### Control variables

Sociodemographic and other control variables were included in the survey distributed at baseline. Sociodemographic variables included the father’s age (in years), the infant’s age (in months, categorized), the father’s origin (Native/Foreign), and household income (in SEK, categorized). Additional control variables included the father’s number of children and the nurse’s invitation of the father to the 3-5-month visit (Yes/No).

#### Nurses’ having answered and asked questions

The independent (exogenous) variables in the main analysis included a scale for nurses’ having answered questions from fathers and a scale for the nurses’ having asked the fathers questions from the father-visit guidelines. These scales were computed from items in the surveys distributed to fathers at baseline. The items related to experiences with the nurse at the home visit, at the 3-5-week visit at the CHC center (later changed to a 1–3 week visit), and at the 3-5-month visit at the CHC center. All items included in the scale for the nurses’ having answered questions (6 items) and in the scale for the nurses having asked questions (17 items) are presented in Appendix 1.

The scale items were originally answered on 7-point Likert scales, but the responses to the items were dichotomized because the distributions of the responses indicated that they were not immediately suitable for inclusion in summative indexes. More specifically, the distributions of the responses were generally U-shaped, reflecting the fact that most responses were either strongly positive or strongly negative, while there were few responses in the intermediate range. To dichotomize the items, responses between 1 and 4 were recoded as 0, and responses between 5 and 7 were recoded as 1. This recoding was in accordance with suggestions previously made for the same items [39]. After dichotomization, the items were summed to produce scales reflecting the extent to which fathers perceived that nurses had asked questions and the extent to which fathers perceived that nurses had answered all of their questions. Finally, the scales were standardized to range between 0 and 1. The standardized scales indicated proportions of items fulfilled by the nurses; for example, the value 0.50 on a scale indicated that the nurse had adhered to half the items that were used to compute the scale in question.

#### Coparenting relationships

Fathers’ coparenting relationships were measured using the brief version of the Coparenting Relationship Scale [44], which has been validated in Sweden [45]. The 12 items for the scale were collected at baseline, at the 6-month follow-up, and at the 18-month follow-up. An example item read, “I believe my partner is a good parent.” The items were answered on 7-point Likert scales. In the current data, the scale had good internal consistency at baseline (Cronbach’s alpha = 0.87), at the 6-month follow-up (Cronbach’s alpha = 0.84), and at the 18-month follow-up (Cronbach’s alpha = 0.85).

### Analysis

The patterns of missingness were analyzed with SPSS. The results of the analysis indicated that the data were not missing completely at random. To reduce the risk of bias due to the patterns of missingness, a multiple imputation of missing data was performed with SPSS.

A correlational analysis of the imputed dataset was performed to assess the effect sizes for the bivariate associations between nurses’ having answered and asked questions and coparenting relationships at each timepoint. The control variables were excluded from the correlational analysis because they were not directly related to the aims of the study. The relationship between infant age and the questions asked and answered by nurses was also examined to account for potential variability due to fathers’ recall of their interactions with nurses. The results indicated no significant associations. The effect sizes were appraised in relation to the conventional guidelines provided by Cohen [46], according to which a small effect size for Pearson’s correlation coefficient *r* is about 0.10, a medium effect size is about 0.30, and a large effect size is about 0.50.

A path model was estimated with IBM SPSS AMOS 21.0 [47]. The model estimated the effect of nurses’ having answered and asked questions and coparenting relationships at each timepoint. Father’s age, the infant’s age, household income, the father’s number of children, the father’s native/foreign origin, pregnancy planning, and the nurse’s invitation of the father to the 3–5 months visit were controlled. We tested the fit of the hypothesized structural model and significance of individual paths. Model fit was assessed using multiple indices: the chi-square test, which indicates adequate fit if non-significant, the root-mean-square error of approximation (RMSEA; values < 0.06 are acceptable), and the Comparative Fit Index (values > 0.95 are acceptable; [48]).

## Results

The descriptive statistics in Table 1 indicated that nurses, on average, fulfilled 79% of the items related to answering fathers’ questions (0.79; 95% CI, from 0.74 to 0.81). By contrast, the nurses fulfilled only 58% of the items related to asking questions from the father visit guidelines (0.58; 95% CI, from 0.44 to 0.51). The difference between the number of items fulfilled in relation to answering and asking questions was significant and large (Cohen’s *d* = 0.87; *p* < .001). The mean values for the coparenting relationships decreased for each successive wave, and compared to baseline, the coparenting relationship means were slightly lower at the 6-month follow-up, which was not significantly different (Cohen’s *d* = 0.06; *p* = .39) and at the 18-month follow-up, which was significantly different (Cohen’s *d* = 0.71; *p* < .001).

The results of the correlational analysis are presented in Table 2. According to the results, all bivariate associations between nurses’ having answered questions, nurses’ having asked questions, and fathers’ coparenting relationships in each wave were significant and had small-to-medium effect sizes (ranging from *r* = .19 to *r* = .26). Also, the correlation between nurses’ having answered questions and nurses’ having asked questions was significant and had a large effect size (*r* = .68), indicating that these variables shared much variance and therefore were likely to reduce each other’s regression parameters and significance levels as they were included as exogenous variables in the same path model.

**Table 2:** Bivariate correlations between the main variables

	Nurse has answered questions	Nurse has asked questions	Coparenting relationship at baseline	Coparenting relationship at 6-month follow-up	Coparenting relationship at 18-month follow-up
Nurse has answered questions	1.00				
Nurse has asked questions	0.68^***^	1.00			
Coparenting relationship at baseline	0.26^***^	0.19^***^	1.00		
Coparenting relationship at 6-month follow-up	0.23^***^	0.23^***^	0.74^***^	1.00	
Coparenting relationship at 18-month follow-up	0.21^*^	0.19^*^	0.59^***^	0.60^***^	1.00

Note. The correlations are based on the imputed datasets. Pearson’s correlation coefficient was used

* p < .05

*** *p* < .001

**Fig. 1:**
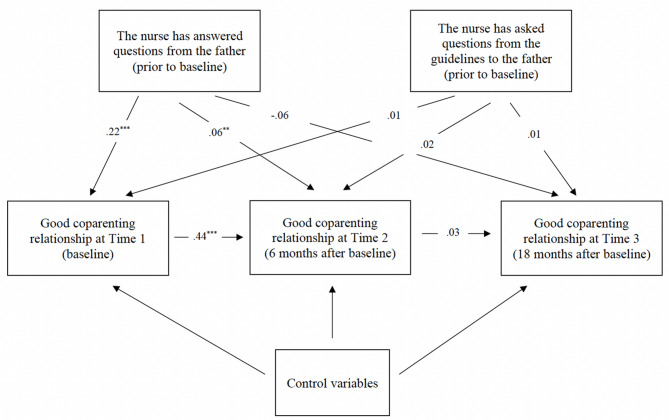
Path model with fathers’ coparenting relationships and nurses’ having answered and asked questions (*n* = 413). The regression parameters are standardized (*β*-coefficients). All covariances between control variables and predictors of interest were estimated but are not displayed in the figure. The control variables include the father’s age, the infant’s age, household income, the father’s number of children, the father’s native/foreign origin, pregnancy planning, and the nurse’s invitation of the father to the 3–5 months visit. The model had excellent fit (*χ*^*2*^_9_ = 8.61, *p* = .47; CFI = 1.00; TLI = 1.00; RMSEA = 0.01 with 90% CI, 0.00 to 0.022). **p < .01. ***p < .001

Figure 1 presents the path model and the parameters of relevance to the study aims. The model had excellent fit (*χ*^2^_9_ = 8.61, *p* = .47; CFI = 1.00; TLI = 1.00; RMSEA = 0.01 with 90% CI, 0.00 to 0.022). Fathers who experienced that the nurses had answered their questions reported better coparenting relationships at baseline (*β* = 0.22, *p* < .001) and at the 6-month follow-up (*β* = 0.06, *p* < .01). There were no other significant results of relevance to the study aims.

## Discussion

The current study investigated whether, when, and to what extent fathers’ coparenting relationships are affected by nurses answering fathers’ questions and by nurses asking fathers’ questions based on the guidelines at the 3–5 month father/non-birthing parent visit at the Swedish CHCs. The primary findings indicate that nurses answering fathers’ questions significantly improved their coparenting relationship at both baseline and the 6-month follow-up, suggesting short- to intermediate-term positive outcomes. However, the effect of nurses asking questions from the guidelines was less pronounced. These results underscore the importance of effective communication and engagement by nurses during early parental visits to foster stronger coparenting relationships.

While nurses in the current study were more likely to engage in answering rather than asking questions, our results highlight that engaging in any form of interaction contributes meaningfully to coparenting support. This underscores the valuable role of nurses in facilitating coparenting dynamics, even if they do not engage in both types of communication equally. However, the results further indicate that nurses who answered fathers’ questions were also more likely to ask them questions, as evidenced by the strong correlation between these behaviours (*r* = .68). This suggests that nurses who engage more actively with fathers tend to both provide and solicit information, reinforcing the importance of comprehensive communication strategies in father-focused child health visits. Since high job performers have a higher level of communication skills, such as providing empathy, adapting communication and managing interactions [49], these findings might indicate that when nurses provide high-quality support, they do so from several angles.

Nurses may not ask as many questions to fathers as they are often more focused and aware of immediate concerns rather than proactively asking questions to uncover underlying issues [50]. However, asking questions and engaging with patients helps promote better patient outcomes [51], leading researchers to call for fundamental organizational changes that bring fathers’ needs into focus [52]. The father/non-birthing parent visit offered at three-to-five months thus helps to promote father involvement; however, more training and support may be needed to ensure that nurses also ask fathers questions and better follow the national guidelines [39]. Asking questions from the guidelines seems to help serve the function of ensuring that fathers and their coparents have the knowledge and abilities they need to maintain a healthy coparenting relationship over time. Enhancing training and resources for nurses to balance both reactive and proactive interactions could potentially further strengthen fathers’ coparenting relationships and help nurses engage fathers more effectively. According to the Swedish child health care guidelines, nurses should ask fathers how their relationship has been affected since having children, how they work together, how they share responsibility for the child and the home, and how they well they think their coparenting relationship works, including how they support each other, work through family conflicts, and how children react to these dialogues [40]. By asking these questions, nurses may provide fathers with ideas on topics to discuss at the CHC (e.g., asking questions) and with their coparent; thus, creating a stronger coparenting relationship. In addition, by providing patient-centered care, nurses help to develop stronger relationships with fathers, which may lead to fathers participating in child health care more often and thus receiving continuous long-term support.

Nurses who answered fathers’ questions effectively contributed to strengthening the coparenting relationship at baseline and at a 6-month follow-up. This suggests that immediate, responsive communication from nurses helps fathers feel supported and understood, which in turn fosters a positive coparenting environment. This finding aligns with previous research highlighting the importance of addressing fathers’ concerns to enhance their involvement and satisfaction in parenting roles. For instance, Daley-McCoy et al. [53] demonstrated that prenatal programs that include fathers and address their specific concerns can reduce psychological distress and improve communication with their partners. Similarly, Premberg and Lundgren [54] found that fathers who felt prepared and supported during prenatal education were more engaged and confident in their parenting roles. Therefore, answering fathers’ questions signals the belief that they are worthy of support, helping them to feel included and involved, leading to improved parental adjustment [43]. Educating and involving fathers early can help them feel more prepared and confident, as well as establish long-term relationships with child health professionals [55]. Therefore, encouraging fathers’ participation in care, such as via implementing a specific fathers/non-birthing parent visit [40], and answering their questions, helps create a supportive environment and promotes positive coparenting interactions.

It is important to note that fathers reported a significant decline in their coparenting relationship at the 18-month follow-up compared to baseline. Additionally, the associations between nurses’ answering fathers’ questions and the coparenting relationship diminished over time. This suggests that early interventions, while beneficial, may not be sufficient to sustain coparenting quality over time. There are at least two transition time periods that might be especially vulnerable for fathers’ coparenting relationships: (i) when fathers start parental leave, and therefore become the primary caregiver, and (ii) when the child enters preschool, and often both parents go to work. Many fathers in Sweden take parental leave when their infant is around a year old, often becoming the primary caregiver to the child [56], while children can only start going to preschool at 12 months of age; meaning that many start after 12 months of age [57]. Thus, during these time periods, fathers may face more difficult and stressful obstacles as they juggle childrearing duties and having a work-life balance, respectively. Since the nurse has a 12-month, 18-month, and 2.5 year check-up with the child, respectively [58], these visits might be an opportune time for nurses to re-examine and further support the coparenting relationship with fathers. Additionally, as the child grows older and parental responsibilities shift, new challenges emerge that require different forms of support. Xiao and Loke [29] emphasize the importance of structured interventions in promoting sustained coparenting cooperation and reducing conflicts. Taken together, this further underscores the importance of continuous engagement with fathers beyond the early infancy stage, potentially through structured follow-ups at later developmental stages.

### Methodological considerations and limitations

A major strength of this study is its longitudinal design, which allowed for the assessment of both short-term and long-term impacts of nurses’ interactions on coparenting relationships. The use of multiple imputation to handle missing data also added robustness to the findings. However, a limitation was the relatively weak significance levels of the main results in the path model. These relatively weaker significance levels were expected, given the multivariate design of the path model. Both nurses’ having answered questions and nurses’ having asked questions were included as exogenous variables in the same path model, and because these variables were highly correlated, they reduced each other’s significance levels. The multiple imputations contributed to making the p-values for the regression parameters even weaker by accounting for and incorporating the uncertainty stemming from missing data patterns in the estimation of regression parameters. However, the main strength of using multiple imputations is the ability to account for and include the uncertainty that stems from missing data, which limits the risk of type I errors based on biased samples.

In the current study, fathers’ age was similar to the national average [59]. Fathers in the current sample had 1.4 children compared to the national average of 1.8 [60]. The current study also had fewer foreign-born fathers (13%) compared to the national average of 19% [61]. And our sample had higher household incomes than the national average [62].

A potential limitation of this study is the recruitment method, which relied on Facebook advertisements. While this approach enabled access to a large and diverse sample of fathers, it may have introduced selection bias. Specifically, Facebook users have been shown to report higher levels of depressive symptoms compared to the general population [63], and our targeted advertisements—particularly those appealing to fathers struggling with parenthood—may have further increased the likelihood of recruiting fathers experiencing distress. As such, the sample may not fully represent the broader population of fathers, particularly those who do not use social media or who are less inclined to engage with parenting-related content online. However, participants in this study reported a wide range of experiences with both coparenting and child health nurse support, suggesting variability in the sample. Moreover, the inclusion of key sociodemographic controls in the analyses strengthens the internal validity of the findings. Therefore, despite the potential for recruitment bias, the results offer meaningful insights that may cautiously generalize to the broader population of Swedish fathers.

Future research should further explore whether answering and asking fathers’ questions within prenatal, perinatal, and pediatric care settings contributes to positive paternal, child, and family outcomes. It will also be important to investigate whether receiving quality child health support early on increases the likelihood of attending subsequent visits, and whether continued support or additional follow-ups help sustain coparenting quality over time.

## Conclusion

This longitudinal study highlights the significant role that nurses play in enhancing fathers’ coparenting relationships through effective communication during early parental visits. Our findings indicate that nurses answering fathers’ questions positively impacts coparenting relationships at both baseline and the 6-month follow-up, underscoring the importance of responsive and supportive interactions early on. However, the less pronounced effect of nurses asking questions suggests a need for more proactive engagement strategies, since nurses often did not completely follow national guidelines. The study also reveals that while early interventions are beneficial, they may not be sufficient to sustain coparenting quality over time, pointing to the necessity for continuous support and follow-ups at later developmental stages. These insights emphasize the critical need for comprehensive training and resources for nurses to balance both reactive and proactive interactions, ultimately fostering a supportive environment that promotes positive coparenting interactions and family well-being. Future research should explore the long-term impacts of such interventions and the potential benefits of structured follow-ups in maintaining coparenting quality.

## Electronic supplementary material

Below is the link to the electronic supplementary material.

Supplementary Material 1

## Data Availability

Due to ethical and legal restrictions related to participant confidentiality, the data are not publicly available. Researchers who meet the criteria for access to confidential data may request access from the corresponding author.
