# Supplementary material for: Nurses’ interactions shape coparenting relationships during early parenthood: a longitudinal study of fathers with infants in Sweden
Source: BMC Nurs. 2025 Oct 1;24:1234. doi: 10.1186/s12912-025-03508-9 (PMC12490136; doi:10.1186/s12912-025-03508-9)
Supplement: Supplementary file 1 — Supplementary Material 1 [file 12912_2025_3508_MOESM1_ESM.docx]

# Appendix 1: Items Used to Compute Scales

## 1. Nurse answered questions

This scale was based on six items with statements related to nurses’ answering questions from fathers. The fathers could respond to the items on 7-point Likert scales, where 1 = “Completely disagree” and 7 = “Completely agree.” The six statements are presented in the table below:

| **Statement** | **Asked in relation to…** |
| --- | --- |
| “I find that there is the opportunity for me to raise questions about being a parent with the CHC nurse.” | All visits, generally |
| “I find that I get answers to my questions from the CHC nurse.” | All visits, generally |
| “I get a good response from the CHC nurse.” | All visits, generally |
| “The CHC nurse answered my questions about being a parent in a way that was supportive and helpful.” | Home visit |
| “The CHC nurse answered my questions about being a parent in a way that was supportive and helpful.” | 3-5-week visit |
| “The CHC nurse answered my questions about being a parent in a way that was supportive and helpful.” | 3-5-month visit |

## 2. Nurse asked questions

This scale was based on 17 items with statements related to the nurses’ asking questions from the guidelines for the father visits. The fathers could respond to the items on 7-point Likert scales, were 1 = “Completely disagree” and 7 = “Completely agree.” The 17 statements are presented in the table below:

| **Statement** | **Asked in relation to…** |
| --- | --- |
| “The CHC nurse asked me how I experienced the birth.” | Home visit |
| “The CHC nurse asked me how my partner and I split the parental responsibility between us.” | Home visit |
| “The CHC nurse asked me how it is possible to combine my work life/student life with my family life.” | 3-5-week visit |
| “The CHC nurse asked me how my partner and I split the parental responsibility between us.” | 3-5-week visit |
| “The CHC nurse asked me if I had started to "get to know" my child.” | 3-5-week visit |
| “The CHC nurse asked me if I was worried about something concerning my child.” | 3-5-week visit |
| “The CHC nurse asked me if I was worried about parenting.” | 3-5-week visit |
| “The CHC nurse asked me about my parental role.” | 3-5-month visit |
| “The CHC nurse asked me about my lifestyle (eg eating habits, alcohol, tobacco, exercise).” | 3-5-month visit |
| “The CHC nurse asked me about my feelings about parenting (eg fear, joy).” | 3-5-month visit |
| “The CHC nurse asked me about my daily routines with my child.” | 3-5-month visit |
| “The CHC nurse asked me how I plan to take my parental leave.” | 3-5-month visit |
| “The CHC nurse asked me how I felt (e.g. mental and physical health).” | 3-5-month visit |
| “The CHC nurse asked if I had enough sleep.” | 3-5-month visit |
| “The CHC nurse asked if I had enough time for my hobbies.” | 3-5-month visit |
| “The CHC nurse asked about the joint parenting with my partner.” | 3-5-month visit |
| “The CHC nurse asked if I had experienced support in my parenting from other people in my vicinity.” | 3-5-month visit |
